# Association of cannabis, cannabidiol and synthetic cannabinoid use with mental health in UK adolescents

**DOI:** 10.1192/bjp.2023.91

**Published:** 2023-10

**Authors:** James Hotham, Rebecca Cannings-John, Laurence Moore, Jemma Hawkins, Chris Bonell, Matthew Hickman, Stanley Zammit, Lindsey A. Hines, Linda Adara, Julia Townson, James White

**Affiliations:** Old Age Psychiatry, Penn Hospital, Black Country Healthcare NHS Foundation Trust, UK; Centre for Trials Research, School of Medicine, Cardiff University, UK; MRC/CSO Social and Public Health Sciences Unit, Institute of Health and Wellbeing, University of Glasgow, UK; Centre for the Development and Evaluation of Complex Interventions for Public Health Improvement (DECIPHer), School of Social Sciences, Cardiff University, UK; Department of Public Health, Environment and Society, London School of Hygiene & Tropical Medicine, UK; Department of Population Health Sciences, University of Bristol, UK; Department of Population Health Sciences, University of Bristol, UK; and MRC Centre for Neuropsychiatric Genetics and Genomics, Cardiff University, UK; Centre for Trials Research, School of Medicine, Cardiff University, UK; and Centre for the Development and Evaluation of Complex Interventions for Public Health Improvement (DECIPHer), School of Social Sciences, Cardiff University, UK

**Keywords:** Anxiety or fear-related disorders, conduct disorders, depressive disorders, substance use disorders, psychotic disorders/schizophrenia

## Abstract

**Background:**

Cannabis has been associated with poorer mental health, but little is known of the effect of synthetic cannabinoids or cannabidiol (often referred to as CBD).

**Aims:**

To investigate associations of cannabis, synthetic cannabinoids and cannabidiol with mental health in adolescence.

**Method:**

We conducted a cross-sectional analysis with 13- to 14-year-old adolescents across England and Wales in 2019–2020. Multilevel logistic regression was used to examine the association of lifetime use of cannabis, synthetic cannabinoids and cannabidiol with self-reported symptoms of probable depression, anxiety, conduct disorder and auditory hallucinations.

**Results:**

Of the 6672 adolescents who participated, 5.2% reported using of cannabis, 1.9% reported using cannabidiol and 0.6% reported using synthetic cannabinoids. After correction for multiple testing, adolescents who had used these substances were significantly more likely to report a probable depressive, anxiety or conduct disorder, as well as auditory hallucinations, than those who had not. Adjustment for socioeconomic disadvantage had little effect on associations, but weekly tobacco use resulted in marked attenuation of associations. The association of cannabis use with probable anxiety and depressive disorders was weaker in those who reported using cannabidiol than those who did not. There was little evidence of an interaction between synthetic cannabinoids and cannabidiol.

**Conclusions:**

To our knowledge, this study provides the first general population evidence that synthetic cannabinoids and cannabidiol are associated with probable mental health disorders in adolescence. These associations require replication, ideally with prospective cohorts and stronger study designs.

Globally, cannabis is the most widely used and internationally regulated illicit drug.^1^ Cannabis use has been consistently associated with an increased risk of mental health disorders. The strongest evidence is found for psychosis,^2^ with limited evidence for depression and anxiety.^3^ Delta-9-tetrahydrocannabinol (THC) is the most researched psychoactive cannabinoid in herbal cannabis.^4^ Experimental evidence suggests that the effects of THC on psychotomimetic symptoms, similar to those experienced with psychosis (e.g. hallucinations, delusions, paranoia), are dose-dependent.^5^ Far fewer studies have researched the effects of other cannabinoids. There has been research suggesting cannabis of a higher potency, defined as the ratio of cannabidiol (often referred to as CBD) to THC, is associated with an increased risk of psychosis and cannabis use disorder,^6^ and some evidence suggesting that cannabidiol attenuates psychotomimetic effects of THC,^7^ but little research into the effects of cannabidiol in isolation. The limited experimental evidence on cannabidiol suggests it may alleviate symptoms of psychosis and social anxiety.^8^ There is a comparable paucity of population-level research into the effects of synthetic cannabinoids. Although THC is a partial agonist with weak affinity for the CB1 receptor, synthetic cannabinoids are full agonists and generally have a higher affinity such that more potent effects might be expected. The limited case reports suggest synthetic cannabinoids can have profound acute effects in young people, including anxiety, paranoia, hallucinations, breathlessness and tachycardia.^9^

## Legal status of cannabis, cannabidiol and synthetic cannabinoids

Cannabis use policy is liberalising in several jurisdictions. The use of the herbal cannabis and associated derived products containing THC is, however, illegal in the majority of the world.^10^ In contrast, synthetic cannabinoids often start as unregulated legal products, but after a period either become controlled (e.g. the USA's Synthetic Drug Abuse Prevention Act of 2012)^11^ or come under blanket acts that ban products with psychoactive effects (e.g. the UK's Psychoactive Substances Act).^12^ In contrast, cannabidiol is a legally marketed product in the USA and many European countries. In the USA in 2019, cannabidiol was one of the top ten selling herbal supplements in mainstream stores, increasing revenue by 872% from 2018, with total USA sales of close to $36 million,^13^ and is often advertised as a natural over-the-counter remedy for mental health problems.^14^

## Methodological limitations of previous research

Nearly all research to date has focused on the association between cannabis and mental health, with very few studies on use of synthetic cannabinoids and cannabidiol. Existing studies have also tended to be either small and thus likely lacking in statistical power, case reviews which might introduce sampling bias,^9^ or based on cannabinoids administered in a laboratory, which is unlikely to reflect how these drugs are used in the general population.^15^ As far as we are aware, there have been no studies of the association between cannabis, synthetic cannabinoids and cannabidiol, and the risk of probable mental health disorders in a general adolescent population sample.

## Aims

To address these limitations, we aimed to (a) describe the use of cannabis, synthetic cannabinoids and cannabidiol among a population of UK adolescents; (b) examine associations between the use of cannabis, synthetic cannabinoids and cannabidiol and symptoms from a range of mental health disorders; and (c) explore the extent to which cannabidiol modifies the association between cannabis and synthetic cannabinoids and probable disorders.

## Method

### Study population

These data arise from baseline assessments within a randomised controlled trial in England and Wales between September 2019 and March 2020.^16^ Schools were randomly sampled from the West of England and South Wales. Questionnaires were self-completed by year 9 students (aged 13–14 years) before random allocation. Fee-paying schools, schools for children only with learning difficulties and pupil referral units were excluded. Paper questionnaires were completed by students in classrooms under examination conditions. Further details are available elsewhere.^16^ Of 7077 eligible students, 6672 participated and completed the questionnaire (94.3% response). The authors assert that all procedures contributing to this work comply with the ethical standards of the relevant national and institutional committees on human experimentation and with the Helsinki Declaration of 1975, as revised in 2008. All procedures were approved by Cardiff University's School of Social Sciences Ethics Committee (approval number: SREC/3342; public trials' registry: ISRCTN72047541). Written informed consent was obtained from all participants and a process of opt-out consent was used with parents. This manuscript adheres to the Strengthening the Reporting of Observational Studies in Epidemiology (STROBE) Statement reporting guidelines.^17^

### Exposure

Participants were asked whether they have ever tried: ‘cannabis (also called: marijuana, spliff, hash, skunk, grass, draw, dab, shatters)’, ‘CBD products (also called: cannabidiol, CBD oil)’ and ‘synthetic cannabinoids (these mimic the effect of cannabis; they are also called: spice, black mamba)’. Images were provided of each type of cannabis reduce misclassification of use (questions shown in the supplementary material available at https://doi.org/10.1192/bjp.2023.91). The images of cannabis were taken from a previously validated measure of cannabis potency,^18^ a stock image was used for cannabidiol and synthetic cannabinoid images were taken from the UK national drug education website (www.talktofrank.com).

### Outcomes

#### Probable depressive disorder

Participants completed the 13-item Short Mood and Feelings Questionnaire, which assesses depressive symptoms over the past 2 weeks.^19^ We applied the validated cut-off point of ≥12 to indicate a disorder.^19^

#### Probable anxiety disorder

Participants completed the seven-item Generalised Anxiety Disorder-7 (GAD-7) screener, which assesses anxiety symptoms over the past 2 weeks.^20^ We applied the validated cut-off point of ≥10, which is considered indicative of a disorder.^20^

#### Probable conduct disorder

The Oregon Adolescent Depression Project Conduct Disorder Screener was completed by participants.^21^ It has six items asking whether participants have engaged in behaviours such as getting into fights in the past week. We applied the cut-off point of ≥9 as indicative of disorder.^21^

#### Auditory hallucinations

A binary measure for experience of an auditory hallucinations (0 indicating no hallucinations, 1 indicating hallucinations) was constructed from self-report data, using questions from the World Health Organization Composite International Diagnostic Interview.^22^ Young people who reported a hallucination were then asked about the distress caused by this experience (response options ranging from not to very distressing) and frequency (not at all to nearly every day/ daily). To increase the diagnostic relevance, we recoded responses into very distressing versus the other categories of distress, and into nearly every day/daily versus the other frequency categories.

#### Covariates

Gender identity was self-reported and categorised as boy, girl or a gender minority (transboy, transgirl, non-binary (neither male nor female), unsure/questioning, other, prefer not to say). Participants also reported their age, ethnicity, employment status of adults they lived with, free school meal entitlement and weekly smoking status (at least one cigarette a week). To describe the characteristics of users and non-users of each cannabinoid, we also assessed participants’ consumption of a whole alcoholic drink in the past 30 days and the frequency of cannabis use over the past 12 months (never, monthly or less, weekly or more).

### Statistical analysis

To describe the characteristics of young people who reported using each type of cannabis, we compared the demographic characteristics, exposure to socioeconomic disadvantage, weekly smoking status and use of alcohol in the past 30 days according to cannabis use, with univariable multilevel logistic regression (students nested within schools). The association between each type of cannabis and mental health was analysed using univariable and multivariable multilevel logistic regression (students nested within schools). Two separate multivariable models were performed for the association of exposure to cannabis, synthetic cannabinoids and cannabidiol with each outcome, to examine the potential confounding effects of (a) adjusting for gender identity and socioeconomic disadvantage (comprising living with no adults in part- or full-time employment and entitlement to free school meals), and (b) after additional adjustment for weekly smoking status. Subgroup analyses were conducted in participants reporting an auditory hallucination into whether use of cannabis, synthetic cannabinoids and cannabidiol were associated with being very distressed by hallucination(s) and hallucinating nearly every day or more. Interactions were then modelled between cannabidiol and both cannabis and synthetic cannabinoids when examining their associations with probable disorders, adjusting for gender identity, socioeconomic disadvantage and weekly smoking status. Results are presented as odds ratios with 95% confidence intervals. To reduce the risk of generating spurious findings resulting from multiple testing, the threshold for significance was Bonferroni-adjusted to *P* < 0.001 (*P* = 0.05/42). Sensitivity analyses were conducted after excluding participants with any missing data and using cannabis frequency as the exposure variable, and examined the percentage overlap in reported cannabis, cannabidiol, synthetic cannabinoid and weekly smoking. Analyses were performed in Stata version 17.0 for Windows (StataCorp).

### Missing data and imputation

Missing data per variable ranged from 1.0 to 9.5%. Missing data in all variables (exposures, outcomes and covariates) were addressed through multiple imputation, using chained equations. Each model included all variables, including the following auxiliary variables: age, ethnicity and alcohol consumption in the past 30 days. Estimates were obtained by pooling results across 20 imputed data-sets according to Rubin rules, and assessment of Monte Carlo errors suggested that this was a suitable number of imputations.^23^

## Results

Of the 6672 participants, 5.2% (95% CI 4.6–5.7%) reported using cannabis, 1.9% (95% CI 1.6–2.3%) reported using cannabidiol and 0.6% (95% CI 0.4–0.7%) reported using synthetic cannabinoids (Table 1). Use of cannabis was more common in participants who identified as a gender minority than boy (12.3 *v*. 5.1%) or girl (4.8%), were entitled to free school meals (9.6 *v*. 4.6%), lived with no employed adults (11.3 *v*. 4.8%), were weekly smokers (79.4 *v*. 4.0%) and had consumed alcohol in the past 30 days (12.0 *v*. 0.9%). Use of cannabidiol was more common in those who were entitled to free school meals (3.9 *v*. 1.6%), lived with no employed adults (3.6 *v*. 1.8%), were weekly smokers (33.3 *v*. 1.8%) and had consumed alcohol in the past 30 days (4.2 *v*. 0.5%). The characteristics of synthetic cannabinoid users were very similar to those for cannabis and cannabidiol. There was a stepped association between cannabidiol and cannabis use in the past 12 months (never, 0.8%; monthly or less, 18.5%; weekly or more, 49.2%) and reported synthetic cannabinoids use (never, 0.1%; monthly or less, 4.7%; weekly or more, 26.7%) (Table 1).

**Table 1:** Association between type of cannabis and participant characteristics (*N* = 6672)

	Cannabis, %			Cannabidiol, %			Synthetic cannabinoids, %		
Characteristic	Never (*n* = 6326)^a^	Used (*n* = 346)	*P*-value^b^	Never (*n* = 6545)	Used (*n* = 127)	*P*-value^b^	Never (*n* = 6632)	Used (*n* = 40)	*P*-value^b^
Gender identity									
Boy	94.9	5.1		97.9	2.1		99.4	0.6	
Girl	95.2	4.8	0.56	98.3	1.7	0.22	99.5	0.5	0.85
Gender minority^c^	87.7	12.3	<0.001	96.7	3.3	0.29	98.6	1.4	0.22
Ethnicity									
White	94.7	5.3		98.1	1.9		99.5	0.5	
Black, Asian and minority ethnic	96.1	3.9	0.17	97.2	2.8	0.14	98.3	1.7	0.001
Entitled to free school meals									
Yes	90.5	9.5		96.1	3.9		98.6	1.3	
No	95.4	4.6	<0.001	98.4	1.6	<0.001	99.5	0.5	0.006
Do not know	94.5	5.5	0.33	97.6	2.4	0.10	99.4	0.6	0.76
Parental unemployment									
Unemployed	88.7	11.3		96.4	3.6		98.5	1.4	
Employed	95.2	4.8	<0.001	98.2	1.8	0.02	99.5	0.5	0.03
Weekly smoking									
Not smoked	96.0	4.0		98.6	1.4		99.7	0.3	
Smoked	20.6	79.4	<0.001	66.7	33.3	<0.001	85.2	14.8	<0.001
Alcohol in past 30 days									
Not consumed	99.1	0.9		99.5	0.5		99.9	0.1	
Consumed	88.0	12.0	<0.001	95.7	4.2	<0.001	98.7	1.3	0.001
12-month frequency of cannabis^d^									
Not used		14.0		99.2	0.8		99.9	0.1	
Monthly or less		65.9		81.5	18.5	<0.001	95.3	4.7	<0.001
Weekly or more		20.0		50.8	49.2	<0.001	73.3	26.7	<0.001

a. All numbers estimated from imputed proportions.

b. Determined by logistic regression.

c. Gender minority comprised transboy, transgirl, non-binary (neither male or female), unsure/questioning and other.

d. No observations on frequency for those who did not use.

The prevalence was 22.7% for probable depressive disorder (95% CI 21.6–23.7%), 19.9% for anxiety disorder (95% CI 18.8–20.9%), 22.1% for conduct disorder (95% CI 21.1–23.1%) and 17.7% (95% CI 16.6–18.8%) for auditory hallucinations. There was a significant unadjusted association between the use of cannabis and probable depressive disorder (odds ratio 3.28, 95% CI 2.58–4.10), anxiety disorder (odds ratio 2.94, 95% CI 2.32–3.72), conduct disorder (odds ratio 8.17, 95% CI 6.35–10.51) and auditory hallucinations (odds ratio 3.10, 95% CI 2.35–4.10) (Table 2). There was also a significant unadjusted association between the use of cannabidiol and probable depressive disorder (odds ratio 3.51, 95% CI 2.40–5.12), anxiety disorder (odds ratio 3.09, 95% CI 2.11–4.53), conduct disorder (odds ratio 8.21, 95% CI 5.38–12.53) and auditory hallucinations (odds ratio 4.65, 95% CI 3.02–7.16). Those who reported using synthetic cannabinoids reported more symptoms consistent with a probable depressive disorder (odds ratio 6.44, 95% CI 3.07–13.52), anxiety disorder (odds ratio 6.24, 95% CI 3.05–12.79), conduct disorder (odds ratio 29.52, 95% CI 10.32–84.42) and auditory hallucinations (odds ratio 12.22, 95% CI 4.29–34.84) than those who had not used. There was little evidence of attenuation of these associations after adjustment for sociodemographic factors. Associations were markedly reduced after the addition of weekly smoking, but participants using any of these cannabinoids remained more than twice as likely to report a probable disorder or an auditory hallucination compared with their peers who reported no use (Table 2).

**Table 2:** Odds ratio for association between lifetime cannabis, cannabidiol and synthetic cannabinoid use with probable depressive disorder, generalised anxiety disorder, conduct disorder and auditory hallucinations (*N* = 6672)

	Odds ratio (95% CI)		
Exposure variable	Unadjusted	Adjusted for sociodemographic factors^a^	Additionally adjusted for weekly smoking
Probable depressive disorder			
Cannabis	3.25 (2.58–4.10)***	3.48 (2.72–4.46)***	2.73 (2.07–3.60)***
Cannabidiol	3.51 (2.40–5.12)***	4.06 (2.71–6.07)***	2.89 (1.87–4.44)***
Synthetic cannabinoids	6.44 (3.07–13.52)***	7.28 (3.36–15.78)***	4.18 (1.80–9.71)
Probable generalised anxiety disorder			
Cannabis	2.94 (2.32–3.72)***	3.00 (2.34–3.85)***	2.35 (1.76–3.13)***
Cannabidiol	3.09 (2.11–4.53)***	3.42 (2.29–5.11)***	2.42 (1.57–3.76)***
Synthetic cannabinoids	6.24 (3.05–12.79)***	6.80 (3.18–14.54)***	4.03 (1.78–9.13)
Probable conduct disorder			
Cannabis	8.17 (6.35–10.51)***	7.88 (6.11–10.16)***	5.60 (4.26–7.35)***
Cannabidiol	8.21 (5.38–12.53)***	7.85 (5.12–12.02)***	5.25 (3.31–8.35)***
Synthetic cannabinoids	29.52 (10.32–84.42)***	28.12 (9.78–80.88)***	16.47 (5.43–49.97)***
Auditory hallucinations^b^			
Cannabis	3.10 (2.35–4.10)***	2.84 (2.13–3.79)***	2.25 (1.62–3.11)***
Cannabidiol	4.65 (3.02–7.16)***	4.57 (2.94–7.12)***	3.44 (2.23–5.55)***
Synthetic cannabinoids	12.22 (4.29–34.84)***	11.00 (3.76–32.19)***	7.08 (2.30–21.85)***

Probable depressive disorder defined as scoring ≥12 on the Short Mood and Feelings Questionnaire; probable generalised anxiety disorder defined as scoring ≥10 on the Generalised Anxiety Disorder-7 screener; probable conduct disorder defined as scoring ≥9 on the Oregon Adolescent Depression Project Conduct Disorder Screener.

a. Sociodemographic factors comprised gender identity, free school meal entitlement and living with an employed parent.

b. Analytical *n* = 5105 as it excludes students who responded that they preferred not to say or did not know whether they had hallucinated.

*** Statistically significant under a Bonferroni-corrected *P* < 0.001 for 42 comparisons.

Among students who reported hallucinations, there was an unadjusted association between the use of cannabis and being very distressed by the hallucination (odds ratio 2.66, 95% CI 1.52–4.65). There was weaker evidence of an association for use of cannabidiol (odds ratio 1.83, 95% CI 0.74–4.52) or synthetic cannabinoids (odds ratio 2.20, 95% CI 0.58–8.33). There was evidence that hallucinating nearly every day was associated with use of cannabidiol (odds ratio 2.15, 95% CI 1.06–4.38) and synthetic cannabinoids (odds ratio 2.88, 95% CI 0.94–8.83), but there was weaker evidence of an association for cannabis use (adjusted odds ratio 1.30, 95% CI 0.71–2.37). Associations were again attenuated after adjusting for weekly smoking (Supplementary Table 1).

There was an interaction between cannabis and cannabidiol use for probable depressive and anxiety disorders. Evidence for an association between cannabis and probable depressive disorder was stronger in those who did not use cannabidiol (adjusted odds ratio 2.77, 95% CI 2.05–3.75) than those who did (adjusted odds ratio 0.66, 95% CI 0.26–1.70; *P*_interaction_ = 0.001). This pattern was also observed in the association between cannabis and probable anxiety disorders (cannabis users: adjusted odds ratio 2.33, 95% CI 1.71–3.18; cannabis non-users: adjusted odds ratio 0.83, 95% CI 0.31–2.18; *P*_interaction_ = 0.001). To explore these interactions, we examined the frequency of cannabis use by cannabidiol use. There was evidence that cannabis use was more frequent in cannabidiol users (never, 40.9%; monthly, 32.7%; weekly, 26.4%) than non-users (never, 96.7%; monthly, 2.8%; weekly, 0.5%).

There was, however, little evidence of an interaction between cannabis and cannabidiol use for symptoms of conduct disorders (*P*_interaction_ = 0.07), auditory hallucinations (*P*_interaction_ = 0.22), nor synthetic cannabinoid and cannabidiol use in associations with probable disorders (depressive disorder: *P*_interaction_ = 0.48; anxiety disorder: *P*_interaction_ = 0.27; conduct disorder: *P*_interaction_ = 0.99; auditory hallucinations: *P*_interaction_ = 0.81).

Sensitivity analysis conducted in the data-sets where there was no missing data showed that the confidence intervals for estimates overlapped with those from the main results using imputed data, indicating there were no meaningful differences (Supplementary Tables 2 and 3). There was a stepwise association between cannabis frequency and outcomes. Adjustment for weekly smoking status attenuated these associations, particularly for those who used cannabis on a weekly basis or more frequently (Supplementary Table 4**)**. There was a greater overlap between weekly smoking with cannabis than cannabidiol or synthetic cannabinoid use (Supplementary Table 5).

## Discussion

### Main findings

In a general population sample of 13- to 14-year-olds in the UK, individuals using cannabis, synthetic cannabinoids or cannabidiol (compared with those who did not) were more likely to report symptoms consistent with a probable depressive disorder, anxiety disorder, conduct disorder or auditory hallucinations. There was some evidence that the strength of associations varied by substance, with the largest associations for synthetic cannabinoids, although confidence intervals for all overlapped. Adjusting for tobacco use attenuated associations, but evidence of these associations was still present. These results are the first to provide a profile of young adolescents who use cannabidiol and synthetic cannabinoids, indicating that, as for cannabis, use is more common among young people who live with parents with a relatively low income, are unemployed, report being a weekly smoker or have used alcohol in the past 30 days. Around 40% of the young people who had used cannabidiol had never used cannabis, whereas around half of those using synthetic cannabinoids had used cannabis weekly or more often over the previous year. As far as we are aware, this is the first study to examine the link between using different cannabinoids and a range of mental health outcomes in adolescence.

### Comparison with previous findings

To our knowledge, the present study gives the first profiles for both cannabidiol and synthetic cannabinoid users in young adolescents. A survey of university students in the USA found that synthetic cannabinoid users did not differ greatly from cannabis users.^24^ Synthetic cannabinoid users were slightly younger than abstainers and more likely to be of non-Hispanic White ethnicity. A household survey of German people aged over 14 years found that having ever used cannabidiol was associated with higher education level, urban living, tobacco use and e-cigarette use.^25^ We replicated the link for cannabidiol and synthetic cannabinoid use with the use of cannabis and tobacco, but found that use of all cannabinoids was associated with greater exposure to household socioeconomic deprivation.

Cannabis use was associated with all of the mental health outcomes assessed. These findings support other reports of cannabis use in adolescents being a risk factor for developing mental illness in early adulthood.^3^ We have characterised the attenuation after adjusting for weekly smoking, because of confounding, as the majority of studies suggest the initiation of smoking tobacco tends to precede cannabis use.^25^ Having said this, recent research indicates the age of tobacco smoking initiation has increased among youth in the USA,^26^ whereas the initiation age for cannabis has remained stable.^27^ There is also data from the UK suggesting that cannabis use has increased over time among 11- to 16-year-olds, whereas tobacco use stayed the same.^28^ This suggests that for some young people, it is possible that regular smoking developed secondary to cannabis use, and that this therefore acted as a mediator rather than a confounding factor, if smoking increases the risk of these disorders. This could mean that this adjusted analysis might underestimate the real associations between cannabis use and mental health outcomes.

Synthetic cannabinoids had large (but imprecise) associations with mental health outcomes. We found fewer than 1% of students had used synthetic cannabinoids. We could not find any other estimates of synthetic cannabinoid prevalence in adolescence. One convenience sample of USA undergraduates found 7.9% reported ever using synthetic cannabinoids, and that use was associated with considering attending an emergency department for anxiety, depression or hallucinations.^24^ Another USA registry study of 107 adolescents presenting to emergency departments found synthetic cannabinoid use was associated with a higher occurrence of acute neuropsychiatric symptoms (e.g. coma, central nervous system depression) than cannabis, and effects were stronger where patients had used other drugs alongside cannabinoids.^29^

Cannabidiol was associated with an increased risk of probable depressive, anxiety and conduct disorders and auditory hallucinations. Randomised and non-randomised controlled trials administering cannabidiol have found improvements in symptoms of psychosis and in patients with social anxiety.^8^ One systematic review of 20 observational studies suggested that cannabis with a higher cannabidiol/THC concentration may have less of an effect on psychosis and cannabis use disorder than cannabis with a lower cannabidiol/THC ratio.^6^ Laboratory studies investigating the effect of cannabidiol consumed before THC or cannabis have had mixed results. One adult study found that people who took cannabidiol before being given THC were less likely to experience clinically significant psychotic symptoms or paranoia,^15^ but other studies with adolescents have not replicated this effect.^30^

In the present study, we found exploratory evidence of an interaction on a multiplicative scale between cannabidiol use and cannabis use, such that the association between cannabis and depression and anxiety was weaker in those who had also used cannabidiol than those who had not. Although these findings are potentially consistent with cannabidiol ameliorating the effects of cannabis on depression and anxiety, there are alternative explanations. It could be that cannabidiol users use cannabis less frequently, have had less cumulative use of cannabis or select cannabis with a higher cannabidiol/THC ratio, compared with those who do not use cannabidiol. We found the frequency of cannabis use was higher in cannabidiol users than non-users, suggesting there is limited support for that explanation. For these reasons, and as we do not know whether individuals ever used cannabidiol and cannabis at the same time, this finding has to be regarded as highly speculative.

### Strengths and limitations

One of the strengths of our study lies in the use of a large sample of adolescents, as both cannabidiol and synthetic cannabinoid have a relatively low prevalence. Another strength is the use of pictorial aids for the different types of cannabis to reduce the chance of misclassification, and multiple validated measures of probable disorders covering a range of symptoms previously linked with cannabis use.^3^ We had a high response rate to our survey and a low amount of missing data for each variable. To account for the missing data, we used multiple imputation to maximise the plausibility of the missing at random assumption. Results were comparable when using the data-sets with missing and imputed data, increasing confidence in the findings. Bias attributable to non-ignorable missing data cannot, however, be ruled out completely.

The cross-sectional design of the study means we cannot establish whether the associations reported are causal. Reverse causality is a tenable explanation for the associations reported, where young people with existing mental health problems choose to use cannabis, synthetic cannabinoids or cannabidiol because they believe it will improve their symptoms. In the case of cannabidiol, this may because cannabidiol is advertised as a natural over-the-counter remedy for mental health problems.^14^ We found an association between use of each cannabinoid and conduct disorder, but it is unclear what mechanism underpins this association. An alternative to a causal explanation is that these associations are brought about by confounding, whereby both cannabinoid use and probable mental health disorder share common antecedents, such as exposure to adverse childhood experiences.^31^ The questions on the use of three cannabinoids did not ask whether use was concurrent. This limits the inferences that can be drawn from the interactions showing that cannabidiol moderated the association between cannabis use and anxiety and depressive symptoms, particularly given the limitation around mechanistic understanding from most patterns of interaction.^32^ It is possible that young people who used both cannabis and cannabidiol had a lower profile of risk factors for mental disorder than those who solely used cannabis. We did not assess the frequency of cannabidiol and synthetic cannabinoid use, limiting inferences that could have been drawn on the extent of exposure. The exclusion of fee-paying schools would have reduced the variability in the assessment of socioeconomic disadvantage and, potentially, the confounding effect of socioeconomic disadvantage on associations.

### Implications

Using data from a general population sample in the UK, we found the use of cannabis, synthetic cannabinoids and cannabidiol was associated with an increased risk of three probable mental health disorders. General population studies are useful in estimating associations at the population level, and can be crucial for informing policy makers and clinical service providers. There is a need for larger trials into the effectiveness and safety of cannabidiol as a treatment for depression, anxiety and psychosis, and greater consideration of how cannabidiol is marketed given the paucity of evidence of benefit to mental health. Our findings signal to clinicians, educators and policy makers that there is a need for education on the uncertainty in the evidence of benefit for cannabidiol and additional research, ideally with longitudinal data into the link between synthetic cannabinoid use and probable mental health disorders in young people.

## Data Availability

The data that support the findings of this study are openly available at https://doi.org/10.17035/d.2023.0244798057.

## Supplementary material

For supplementary material accompanying this paper visit https://doi.org/10.1192/bjp.2023.91. click here to view supplementary material
