# Supplementary material for: Association of cannabis, cannabidiol and synthetic cannabinoid use with mental health in UK adolescents
Source: Br J Psychiatry. 2023 Oct;223(4):478–84. doi: 10.1192/bjp.2023.91 (PMC10866672; doi:10.1192/bjp.2023.91)
Supplement: Supplementary file 1 [file S0007125023000910sup001.docx]

**Supplementary materials**

**Questions used to assess cannabis, CBD and synthetic cannabinoid use**

Below is a list of different types of drugs and some of their street names. This question asks if you have **EVER TRIED** any drugs. **In your life have you ever TRIED any of the following?**

(Please mark an **X** for **YES** I have tried or **NO** I have **NOT** tried for **EVERY** drug listed below)

For these first three drugs we want you to think carefully about how they are different from each other. This is because sometimes people can be confused about which one is which, and so it’s important you read each question to yourself first before you give your answers.


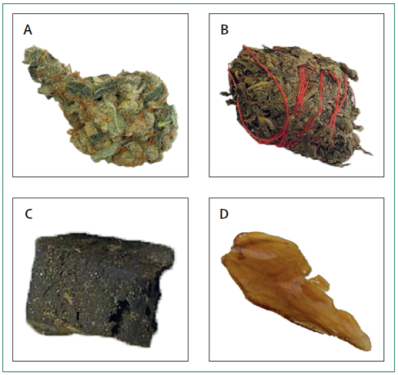
**a) Cannabis** (also called: marijuana, weed, spliff, hash, skunk, grass, draw, dab, shatters):

Yes I have tried No I have not tried


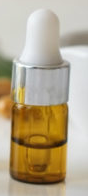
**b) CBD Products** (also called: cannabidiol, CBD oil):

Yes I have tried No I have not tried


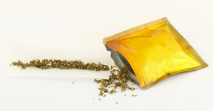
**c) Synthetic cannabinoids** (these mimic the effect of cannabis: also called: spice, black mamba, mary joy):

Yes I have tried No I have not tried

Table S1. Odds ratio (95% confidence interval) for association between lifetime cannabis, cannabidiol and synthetic cannabinoid use with being very distressed and hearing a hallucination nearly every day in subgroup who reported an auditory hallucination (n = 905)

|  | **Very distressed by** **an auditory hallucination** | | | | |
| --- | --- | --- | --- | --- | --- |
| **Exposure variable** | **Unadjusted** | **Adjusted for sociodemographic factors** ^a^ | **Additionally adjusted for weekly smoking** | |  |
| Cannabis | 2.66 (1.52, 4.65) | 2.47 (1.40, 4.36) | 2.81 (1.45, 5.44) | |  |
| Cannabidiol | 1.83 (0.74, 4.52) | 1.95 (0.78, 4.91) | 1.89 (0.69, 5.21) | |  |
| Synthetic cannabinoids | 2.20 (0.58, 8.33) | 2.42 (0.63, 9.33) | 2.25 (0.52, 9.76) | |  |
|  |  |  |  | |  |
|  | **Auditory hallucination nearly every day** | | |  |  |
| Cannabis | 1.30 (0.71, 2.37) | 1.16 (0.63, 2.14) | 0.82 (0.37, 1.81) | |  |
| Cannabidiol | 2.15 (1.06, 4.38) | 2.24 (1.09, 4.61) | 1.89 (0.87, 4.09) | |  |
| Synthetic cannabinoids | 2.88 (0.94, 8.83) | 3.13 (1.00, 9.81) | 2.44 (0.72, 8.31) | |  |

^a^ Sociodemographic factors comprised: gender identity, free school meal entitlement and living with an employed parent.

|  | **Probable depressive disorder** | | | | | |  |
| --- | --- | --- | --- | --- | --- | --- | --- |
| **Exposure variable** | **Unadjusted** | **Adjusted for sociodemographic factors** ^a^ | **Additionally adjusted for weekly smoking** | |  |  |  |
| Cannabis | 4.10 (2.91, 5.77) | 4.16 (2.89, 5.99) | 3.01 (1.99, 4.55) | |  |  |  |
| Cannabidiol | 4.23 (2.44, 7.35) | 4.58 (2.55, 8.25) | 3.01 (1.60, 5.66) | |  | | |
| Synthetic cannabinoids | 23.84 (5.12, 110.93) | 24.92 (5.11, 121.56) | 10.75 (2.00, 57.78) | |  |  |  |
|  |  |  |  | |  |  |  |
|  | **Probable generalized anxiety disorder** | | |  |  |  |  |
| Cannabis | 4.11 (2.91, 5.80) | 4.04 (2.81, 5.81) | 3.35 (2.22, 5.05) | |  |  |  |
| Cannabidiol | 4.63 (2.67, 8.04) | 4.88 (2.71, 8.76) | 3.58 (1.93, 6.64) | |  |  |  |
| Synthetic cannabinoids | 15.67 (4.17, 58.82) | 15.57 (3.94, 61.61) | 7.75 (1.83, 32.79) | |  |  |  |
|  |  |  |  | |  |  |  |
|  | **Probable conduct disorder** | | | | |  |  |
| Cannabis | 9.51 (6.66, 13.58) | 9.30 (6.50, 13.32) | 6.05 (4.09, 8.94) | |  |  |  |
| Cannabidiol | 9.40 (5.25, 16.84) | 8.78 (4.87, 15.80) | 5.75 (3.02, 10.91) | |  |  |  |
| Synthetic cannabinoids | 53.82 (6.85, 422.85) | 53.40 (6.72, 424.18) | 23.90 (2.59, 220.47) | |  |  |  |
|  |  | | | | |  |  |
|  | **Auditory hallucinations** | | | | |  |  |
| Cannabis | 3.67 (2.59, 5.19) | 3.38 (2.37, 4.82) | 2.39 (1.58, 3.62) | |  |  |  |
| Cannabidiol | 7.41 (4.24, 12.96) | 6.98 (3.94, 12.37) | 5.12 (2.79, 9.38) | |  |  |  |
| Synthetic cannabinoids | 27.91 (6.01, 129.57) | 25.83 (5.46, 122.28) | 12.20 (2.35, 63.45) | |  |  |  |

Table S2. Odds ratio (95% confidence interval) for association between lifetime cannabis, cannabidiol and synthetic cannabinoid use with probable depressive disorder, generalised anxiety disorder, conduct disorder and auditory hallucinations in the sample with no missing data (n = 3491)

^a^ Sociodemographic factors comprised: gender identity, free school meal entitlement and living with an employed parent.

Table S3. Odds ratio (95% confidence interval) for association between lifetime cannabis, cannabidiol and synthetic cannabinoid use with probable depressive disorder, generalised anxiety disorder, and conduct disorder in individuals reporting an auditory hallucination in the sample with no missing data (n = 447)

|  | **Very distressed by** **an auditory hallucination** | | | |
| --- | --- | --- | --- | --- |
| **Exposure variable** | **Unadjusted** | **Adjusted for sociodemographic factors** ^a^ | **Additionally adjusted for weekly smoking** | |
| Cannabis | 2.83 (1.38, 5.81) | 2.79 (1.35, 5.81) | 3.19 (1.38, 7.36) | |
| Cannabidiol | 1.37 (0.43, 4.35) | 1.41 (0.43, 4.64) | 1.23 (0.34, 4.47) | |
| Synthetic cannabinoids | 4.20 (0.90, 19.52) | 4.02 (0.86, 18.83) | 4.06 (0.69, 23.94) | |
|  |  |  |  | |
|  | **Auditory hallucination nearly every day** | | |  |
| Cannabis | 1.36 (0.66, 2.79) | 1.26 (0.60, 2.63) | 0.65 (0.23, 1.79) | |
| Cannabidiol | 3.48 (1.42, 8.51) | 3.20 (1.25, 8.18) | 2.29 (0.82, 6.38) | |
| Synthetic cannabinoids | 8.64 (2.02, 36.96) | 8.30 (1.87, 36.85) | 5.45 (1.07, 27.66) | |

^a^ Sociodemographic factors comprised: gender identity, free school meal entitlement and living with an employed parent.

Table S4. Odds ratio (95% confidence interval) for association between the frequency of cannabis use over the past 12 months with probable depressive disorder, generalised anxiety disorder, conduct disorder and auditory hallucinations (n = 6672)

|  | **Probable depressive disorder** | | |
| --- | --- | --- | --- |
| **Exposure variable** | **Unadjusted** | **Adjusted for sociodemographic factors ^a^** | **Additionally adjusted for weekly smoking** |
| Never | 1.00 (Reference) | - | - |
| Monthly or less | 3.12 (2.34, 4.16) | 3.21 (2.35, 4.38) | 2.67 (1.93, 3.71) |
| Weekly or more | 4.27 (2.54, 7.18) | 4.91 (2.81, 8.57) | 2.68 (1.37, 5.22) |
|  |  |  |  |
|  | **Probable generalized anxiety disorder** | | |
| Never | 1.00 (Reference) | - | - |
| Monthly or less | 3.07 (2.30, 4.10) | 3.06 (2.56, 4.16) | 2.49 (1.78, 3.47) |
| Weekly or more | 2.85 (1.68, 4.82) | 3.00 (1.71, 5.25) | 1.49 (0.75, 2.95) |
|  |  |  |  |
|  | **Probable conduct disorder** | | |
| Never | 1.00 (Reference) | - | - |
| Monthly or less | 7.74 (5.65, 10.59) | 7.52 (5.49, 10.31) | 5.90 (4.23, 8.22) |
| Weekly or more | 11.57 (6.42, 20.85) | 11.00 (6.06, 20.00) | 4.01 (2.04, 7.87) |
|  |  | | |
|  | **Auditory hallucinations** | | |
| Never | 1.00 (Reference) | - | - |
| Monthly or less | 2.88 (2.03, 4.08) | 2.60 (1.81, 3.71) | 2.18 (1.48, 3.22) |
| Weekly or more | 4.22 (2.27, 7.82) | 3.86 (2.05, 7.28) | 2.20 (1.04, 4.62) |

^a^ Sociodemographic factors comprised: gender identity, free school meal entitlement and living with an employed parent.

Table S5. Percentage overlap between each type of cannabis and weekly smoking (n = 6672)

|  | **Cannabis** |  | **CBD** |  | **Synthetic cannabinoids** | | **Weekly smoking** | |
| --- | --- | --- | --- | --- | --- | --- | --- | --- |
|  | **Never** | **Used** | **Never** | **Used** | **Never** | **Used** | **Never** | **Used** |
| **Cannabis** |  |  |  |  |  |  |  |  |
| Never | 100 | 0 | 99.2 | 0.8 | 99.9 | 0.1 | 99.7 | 0.3 |
| Used | 0 | 100 | 77.0 | 23.0 | 90.6 | 9.4 | 76.3 | 23.7 |
| **CBD** |  |  |  |  |  |  |  |  |
| Never | 95.9 | 4.1 | 100 | 0 | 99.7 | 0.3 | 99.0 | 1.0 |
| Used | 38.5 | 61.5 | 0 | 100 | 84.5 | 15.5 | 73.5 | 26.5 |
| **Synthetic cannabinoids** |  |  |  |  |  |  |  |  |
| Never | 95.3 | 4.7 | 98.4 | 1.6 | 100 | 0 | 98.7 | 1.3 |
| Used | 13.5 | 86.5 | 46.4 | 53.6 | 0 | 100 | 59.2 | 40.8 |
| **Weekly smoking** |  |  |  |  |  |  |  |  |
| Never | 96.0 | 4.0 | 98.6 | 1.4 | 99.7 | 0.3 | 100 | 0 |
| Used | 20.6 | 79.4 | 66.7 | 33.3 | 85.2 | 14.8 | 0 | 100 |
